# Inflammation-related signaling pathways in tendinopathy

**DOI:** 10.1515/biol-2022-0729

**Published:** 2023-09-20

**Authors:** Li Jiang, Tianzhu Liu, Kexin Lyu, Yixuan Chen, Jingwei Lu, Xiaoqiang Wang, Longhai Long, Sen Li

**Affiliations:** School of Physical Education, Southwest Medical University, Luzhou, 646000, China; Neurology Department, The Affiliated Traditional Chinese Medicine Hospital of Southwest Medical University, Luzhou, 646000, China; The Affiliated Traditional Chinese Medicine Hospital of Southwest Medical University, Luzhou, 646000, China; Division of Spine Surgery, Department of Orthopedic Surgery, Nanjing Drum Tower Hospital, Affiliated Hospital of Medical School, Nanjing University, Nanjing, 210000, China

**Keywords:** inflammation, signaling pathway, tendinopathy, tendon repair

## Abstract

Tendon is a connective tissue that produces movement by transmitting the force produced by muscle contraction to the bones. Most tendinopathy is caused by prolonged overloading of the tendon, leading to degenerative disease of the tendon. When overloaded, the oxygen demand of tenocytes increases, and the tendon structure is special and lacks blood supply, which makes it easier to form an oxygen-deficient environment in tenocytes. The production of reactive oxygen species due to hypoxia causes elevation of inflammatory markers in the tendon, including PGE2, IL-1β, and TNF-α. In the process of tendon healing, inflammation is also a necessary stage. The inflammatory environment formed by cytokines and various immune cells play an important role in the clearance of necrotic material, the proliferation of tenocytes, and the production of collagen fibers. However, excessive inflammation can lead to tendon adhesions and hinder tendon healing. Some important and diverse biological functions of the body originate from intercellular signal transduction, among which cytokine mediation is an important way of signal transduction. In particular, NF-κB, NLRP3, p38/MAPK, and signal transducer and activator of transcription 3, four common signaling pathways in tendinopathy inflammatory response, play a crucial role in the regulation and transcription of inflammatory factors. Therefore, summarizing the specific mechanisms of inflammatory signaling pathways in tendinopathy is of great significance for an in-depth understanding of the inflammatory response process and exploring how to inhibit the harmful part of the inflammatory response and promote the beneficial part to improve the healing effect of the tendon.

## 1 Introduction

The composition of the tendon is more complex, and it is a dense hoof tissue mainly composed of collagen type I [1]. Therefore, it can withstand larger loads and has strong tensile strength. The forces generated by the muscles are transmitted through the tendons to the bones, which result in various movements [2]. However, tendons are prone to pathological changes due to some intrinsic factors such as age, metabolic diseases, or when the tendons are subjected to repetitive mechanical loads [3,4]. The pathology of tendinopathy is manifested in many ways. The extracellular matrix (ECM) becomes disorganized, and the type III collagen content is increased, resulting in a decrease in the tendon’s ability to resist stretch. There is also a large accumulation of proteoglycans and glycosaminoglycans, as well as a proliferation of cells and the formation of neovascularization [5]. Tendon repair, on the other hand, is a lengthy process that typically involves three overlapping stages: inflammation, proliferation, and remodeling [6]. The role of inflammation in tendinopathy has been highly debated. Initially, researchers believed that inflammation was the primary pathological change following a tendon injury and that inflammation could lead to pain and functional impairment. During this period, the term “tendonitis” was generally accepted [7]. Subsequent studies showed that no infiltration of inflammatory cells such as macrophages was found at the site of tendon injury, so the concept of “tendinitis” was abandoned [8]. In recent years, with the advancement of pathology and immunology, studies have demonstrated the presence of macrophages, T cells, and B cells in chronic tendinopathy using monoclonal antibodies [9]. At the same time, more and more studies have also shown that inflammation plays an important role in the development of tendinopathy [10].

The inflammatory response is a critical step in wound healing because only by removing necrotic tissue and waste products through the inflammatory response [11], and then activating tissue-resident cells and stimulating their conversion into myofibroblasts, can the proliferative phase begin and tendon repair can proceed smoothly [12]. Nuclear factor kappa beta (NF-κB) is an important transcription factor that regulates inflammation. After a tendon injury, NF-κB usually acts as a heterodimer formed by the combination of p50 and p60 [13] and promotes inflammatory mediators and chemokines such as Interleukin-1β (IL-1β) and Interleukin-6 (IL-6), tumor necrosis factor (TNF-α), chemokines CCL2, CXCL10, and other releases [14]. At the same time, some other important inflammatory signaling pathways such as NLRP3, p38/Mitogen-activated protein kinases (MAPK) and signal transducer and activator of transcription 3 (STAT3) are also activated during the inflammatory stage. The factors that activate these signaling pathways and the inflammatory responses generated through the signaling pathways are described in Table 1. The inflammatory cascade generated by the above-mentioned signaling pathways drives the expression of pro-inflammatory genes and further promotes the remodeling of damaged tissues to restore homeostasis [15,16,17]. However, a persistent inflammatory response activates the fibroblast population and promotes excessive deposition of matrix, ultimately leading to the formation of scar tissue [18]. Therefore, a controlled inflammatory response is critical to the overall process of tendon healing.

**Table 1:** Factors that activate inflammatory signaling pathways and the resulting inflammatory response

Signaling pathway	Inducing factors	Inflammatory responses
NF-κB	IL-1β, TNF-α	These inducers promote the growth and development of B cells and the co-stimulatory effect of T cells. They also upregulate the expression of inflammatory factors, including IL-8, IL-1β, TNF-α, COX-2, IL-6 and MIP-2
	LTβR, BAFFR, CD40, RANK	
	Hypoxia, HIF-1	
NLRP3	HMGB1,ROS	Promoting the secretion of IL-1β, IL-18, TNF-α, and IL-6
	Reduction of COL1:COL3 ratio	
	Adipose tissue infiltration	
	Change in ion concentration	
	(K^+^, Ca^2+^, Cl^−^)	
p38/MAPK	Inflammatory factors, growth factors, or environmental stress (e.g., oxidative stress, DNA damage, mechanical loading)	Promoting the production of inflammatory factors such as TNF-α, IL-6, and IL-8 by monocytes and macrophages
STAT3	Interferons, interleukins, growth factors	Increasing the expression of IL-6 and IL-10

Therefore, the purpose of this review is to elucidate the mechanisms of the major inflammatory signaling pathways during tendon healing and to further explore how to cut off the harmful part of the inflammatory response and preserve the beneficial part. It is also hoped that the inflammatory response can be promoted in a manageable direction, reducing the formation of scar tissue and offering new hope for the treatment of tendinopathies.

**Figure 1:**
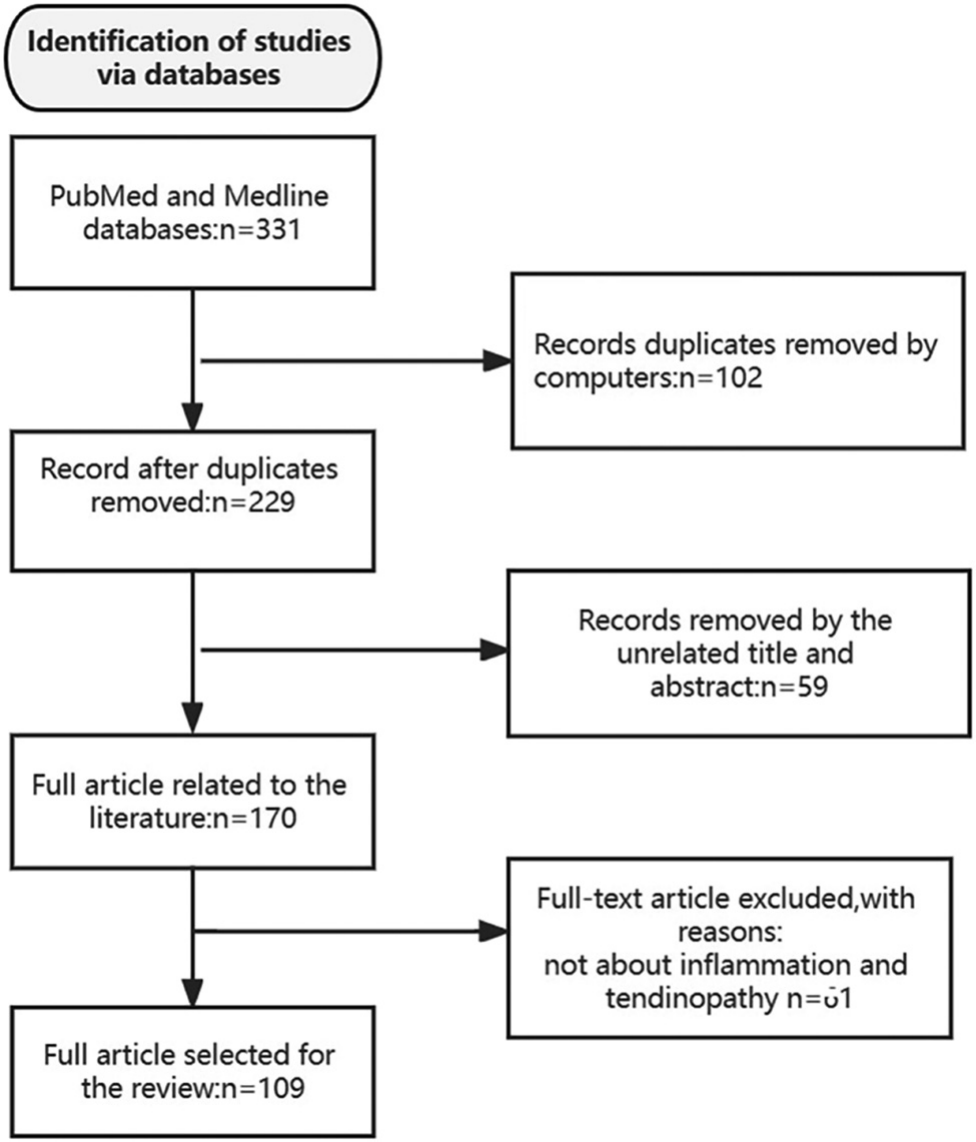
Article retrieval flow chart with inclusion and exclusion processes.

**Search strategy**. (i) Search site: articles are from PubMed, a database of articles on biomedical science. (ii) Database: MEDLINE. (iii) Keywords: inflammation, signaling pathway, tendinopathy, and tendon repair. (iv) Boolean algorithm: (“inflammation” OR “signaling pathway”) AND (“tendinopathy” OR “tendon healing” OR “tendon repair”). (v) Retrieval timeframe: We searched the selected journals from 2000 to 2023. (vi) Inclusion and exclusion criteria: articles were included if their topic was related to inflammation and tendinopathy, and if the article type was a review or an experimental article. The search process was performed as presented in Figure 1.

## 2 Inflammatory response is the beginning of tendinopathy

**Figure 2:**
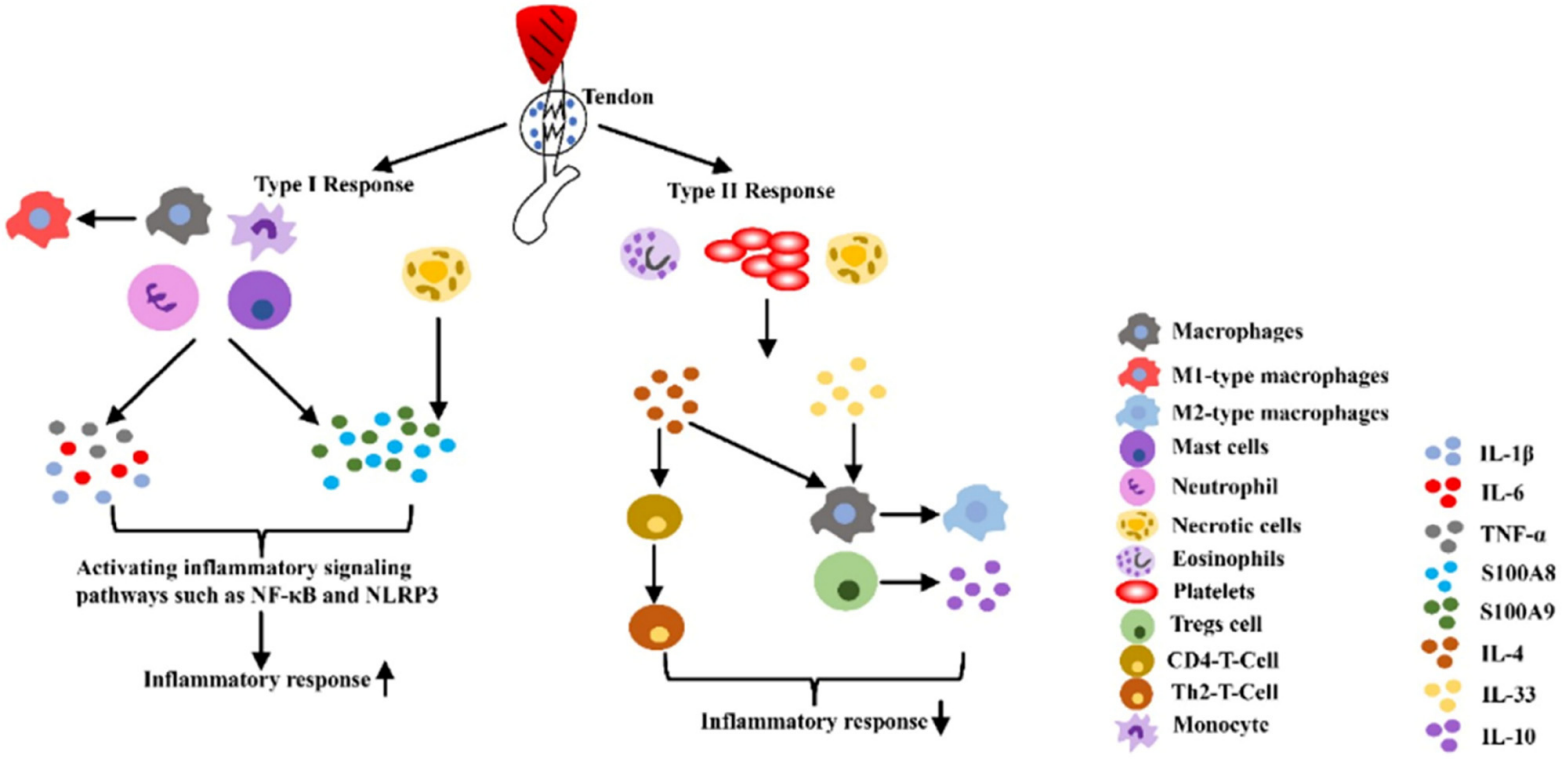
Two immune response processes in the inflammatory phase of tendinopathy.

After a tendon injury, the injury site may show signs of pain, exudation, redness, and dysfunction [19]. Although earlier studies have shown that tendon injury is a degenerative disease caused by excessive use of tendons and no inflammatory cells were found, as the research has progressed, there is mounting evidence that inflammatory factors play an important role following tendon injury [10]. After a tendon injury, its reparative healing goes through three phases: the inflammatory phase, the proliferative phase, and the remodeling phase [6]. The inflammatory phase first removes necrotic cells and creates a temporary extracellular matrix to promote cellular replenishment. Next, the immune system begins to recruit immune cells and secretes cytokines that stimulate cell proliferation and tissue remodeling. These inflammatory responses are directed by distinct type I (pro-inflammatory) and type II (anti-inflammatory) immune regimens (Figure 2) [20]. In the type I immune response, S100A8 and S100A9 act as alarm elements that are released into the extracellular environment by necrotic cells or activated immune cells [21]. This then leads to enhanced recruitment of immune cells (Th1 T cells, neutrophils, and M1-type macrophages) and promotes the release of pro-inflammatory factors such as TNF-α, IFN-γ, IL1-β, and iNOS from tendon cells [22]. Subsequently, downstream inflammatory signaling pathways such as NF-κB and NLRP3 are activated, regulating inflammatory genes as well as transcription [23,24]. Furthermore, the presence of pro-inflammatory factors breaks down the ECM and promotes new ECM deposition [25]. In order to prevent the excessive pro-inflammatory response of the type I immune response, the body activates the type II immune response for anti-inflammation by secreting IL-4 or IL-33 from damaged cells [26]. The release of IL-33 triggers downstream responses from macrophages, Tregs, and other intrinsic immune cells [27]. In particular, Tregs can produce IL-10, which acts as an important anti-inflammatory factor to resolve inflammation in the type I immune response. IL-4 can also promote the conversion of naive CD4 T cells and macrophages to Th2 T cells and M2-type macrophages, thus exerting anti-inflammatory effects [28].

IL-1 is an important inflammatory cytokine in the inflammatory response [29]. It plays an important role in degrading the extracellular matrix, inhibiting tendon cell markers, and inducing pain. After a tendon injury, inflammatory factors such as IL-1 and TNF-α are released by inflammatory cells such as neutrophils and macrophages during the exogenous healing phase [30]. IL-1β downregulates the gene expression of early growth response gene 1 (Egr1), Col1, and Col3, while upregulating the expression of matrix metalloproteinases 1, 3, 8, and 13 (MMP1, 3, 8, and 13) [31]. MMPs mediate the catabolism of collagen, leading to sustained tissue degradation [32]. In addition, IL-1β downregulates the expression of the tendon cell markers SCX and TNMD, which leads to a decrease in the ultimate tensile strength and elastic modulus of the repaired tendon [33]. In damaged tissues, PGE2 acts to promote vasodilation and elicit a pain hypersensitivity response. It was shown that IL-1β accelerates the conversion from PGH2 to PGE2 and causes an increase in perceived pain by enhancing the expression of prostaglandin E synthase (mPGES) [34]. All of this evidence suggests that IL-1 plays an important role in the development of the inflammatory response. After IL-1 and TNF-α are released, they bind to TLR4 on the cell membrane [35]. The polymerization of TLR4 enables signal transduction into cells; there is a TIR region (Toll/IL-1 Receptor region) in the cell membrane of TLR4 that binds to the carboxy terminus of MyD88 [36]. At the same time, the amino-terminal death domain of MyD88 binds to the amino-terminal death domain of IL-1 receptor-associated kinase (IRAK). This process promotes the phosphorylation of IRAK and the acquisition of free IRAK1, IRAK2, and IRAK4, which in turn activates TNF-α receptor-associated factor 6 (TRAF-6). Next, TRAF-6 binds to NF-κB kinase and phosphorylates the beta subunit of NF-κB kinase (IKKβ), thereby activating the IκB kinase (IKK) complex [37]. IKK induces IκB phosphorylation at residues Ser32 and Ser36 of IκBα and residues Ser19 and Ser23 of IκBβ through the 26 S proteasome [38]. IκB is subsequently degraded, which results in the release of NF-κB activity and the entry of p50-p65 into the nucleus to initiate the expression of downstream genes regulated by NF-KB [39,40]. NF-κB acts as a powerful pro-inflammatory signaling pathway that drives the production of many pro-inflammatory cytokines, including IL-1, IL-6, CCL2, and TNF-α. In turn, these inflammatory can reactivate NF-κB activity, so there is often a persistent inflammatory response during tendon healing [41]. The persistent inflammatory environment has a negative impact on tendon healing and also leads to the formation of tissue adhesions during the collagen remodeling phase [13].

The inflammatory phase after tendon injury is completed by type I and type II immune responses. Type I immune responses activate downstream inflammatory signaling pathways primarily through the release of pro-inflammatory factors from necrotic cells and immune cells. Type II immune response is mainly through the secretion of anti-inflammatory factors by damaged cells, which continue to activate other immune cells and promote the secretion of anti-inflammatory factors.

## 3 Role of NF-κB signaling pathway

**Figure 3:**
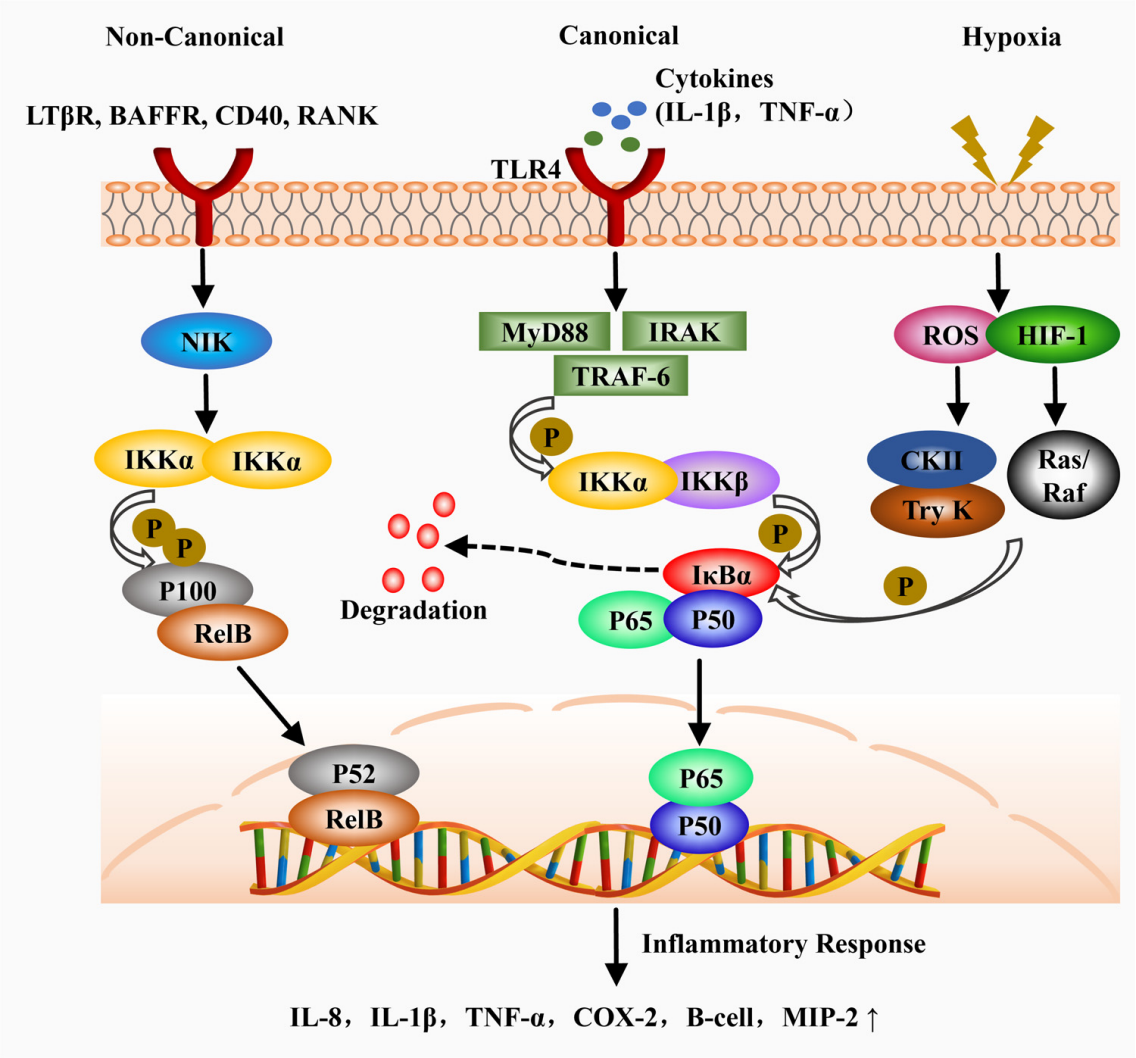
NF-κB-Mediated inflammatory response process.

After tenocyte injury, the blood forms a clot at the injury site, and platelets subsequently release large amounts of growth factors and cytokines. At the same time, platelets play a chemotactic role, attracting leukocytes, macrophages, and neutrophils to aggregate at the injury site and phagocytose to remove necrotic material [1]. Extrinsic healing and early inflammatory responses are initiated at this point. During the inflammatory response stage, some signaling pathways are activated in coordination, and the expression of pro-inflammatory and anti-inflammatory mediators is regulated, thereby promoting the homeostasis of the ECM [42]. NF-κB is present at all stages of tendon healing, involving processes such as inflammation, cell proliferation, angiogenesis, and formation of tissue adhesions [43]. In the inflammatory stage, NF-κB is a strong pro-inflammatory signaling pathway, and NF-κB mainly plays a role in the expression of pro-inflammatory genes including cytokines, chemokines, and adhesion molecules [44]. The phase of the inflammatory response driven by the NF-κB signaling cascade greatly affects the balance of the inflammatory response and the outcome of tissue healing [45]. Tendon repair is carried out in two ways, including endogenous healing and exogenous healing. Endogenous healing mainly consists of tenocytes proliferating, migrating, and completing collagen synthesis under the action of cytokines. Exogenous healing is an important part of tendon healing, which is mainly completed by fibroblasts and inflammatory cells [46]. Inflammatory factors such as IL-1β and TNF-α are released by inflammatory cells such as neutrophils and macrophages after tendon injury [30]. Under the stimulation of these inflammatory factors, IκB is phosphorylated through a variety of signal transduction pathways and then degraded under the action of proteolytic enzymes, thereby activating NF-κB signaling and exerting its regulatory effect on the inflammatory response. The above-mentioned process is the activation pathway of canonical NF-κB [23]. In addition, NF-κB can also be activated through a non-classical pathway induced by CD40 and the hypoxic environment that results from tendon injury [47,48]. The process of NF-κB involved in the inflammatory response is described in detail in Figure 3.

NF-κB, as a classical inflammatory response signaling pathway, has been studied in depth. Meanwhile, studies on the inhibition of NF-κB pathway activity are also more extensive. IKKβ serves as a key subunit that promotes NF-κB activation, and selective inhibition of its activity may be an important way to attenuate the inflammatory response [45]. In a study on tendon repair in dogs, it was shown that after oral administration of the IKKβ inhibitor ACHP, a reduction in the extent of phosphorylated p65 (p-p65) was demonstrated on day 14, along with suppression of the expression of inflammation-related genes. In addition, cell proliferation was significantly enhanced and neovascularization increased, suggesting that targeted inhibition of NF-κB activity can reduce inflammation in the early stages of tendon repair while accelerating the tendon repair process[49]. Similarly, the application of ACHP in a rat rotator cuff injury model showed favorable therapeutic effects. It was administered orally once a day for 7 days after rotator cuff surgery in rats. The results showed a significant reduction in the expression of inflammation-related genes in the NF-κB pathway, and enhanced extracellular matrix production was observed at the injury site. The results observed after stimulation of rat tendon fibroblasts using ACHP were also the same as those in the *in vivo* experiments, showing satisfactory results[50]. In addition to the application of ACHP to inhibit the activity of NF-κB, there are several other targeted inhibitors of NF-κB that have also shown good results in experiments. For example, the addition of two different p65-specific inhibitors, Helenalin and JSH23, respectively, to cultured fibroblasts showed that the protein expression of p-p65, Type I collagen (COL I), Type Ⅲ collagen (COL III), α-SMA, and COX-2 could be significantly reduced. Transfections of NIH3T3 fibroblasts and fibroblast with p65-siRNA, which selectively inhibits p65, showed a significant reduction in the expression of p65, as well as a decrease in the expression of other inflammatory factors. Although the effects of the three inhibitors were similar, there were some differences in their mechanisms of inhibition. For example, p65-siRNA inhibits p65 gene expression by specifically degrading p65 mRNA and thus inhibiting p65. Whereas Helenalin selectively alkylated the p65 subunit, JSH23 prevented the translocation of NF-κB into the nucleus[51]. Despite the different approaches, all of them could reduce the inflammatory response by inhibiting NF-κB activity. In addition to specific inhibitors of NF-κB, Calebin A (CA), a natural drug, was found to have similar effects to BMS-345541 (specific IKK inhibitor)[52]. It can have the effect of inhibiting the activity of NF-κB by inhibiting the phosphorylation and degradation of IκBα. This has been demonstrated in *in vitro* experiments on tenocytes [53].

NF-κB can be involved in the inflammatory response through canonical and non-canonical pathways as well as induced by hypoxia.

### 3.1 The canonical NF-κB activation pathway

NF-κB is an inducible transcription factor family consisting of five structurally related members, including NF-κB1 (also known as p50), NF-κB2 (also known as p52), RelA (also known as p65), RelB and c-Rel, mediate the transcription of target genes in the form of various heterodimers or homodimers by binding to specific DNA sites [54,55]. NF-κB is a typical heterodimer, and the most common structure is a complex composed of the proteins p50 and p65. In the inactive state, the activity of NF-κB is inhibited by the protein inhibitor IκB and retained in the cytoplasm, preventing the activated NF-κB from moving into the nucleus and regulating downstream gene transcription [48]. NF-κB has long been recognized as a canonical inflammatory signaling pathway that mainly responds to stimulation by proinflammatory cytokines such as IL-1 and TNF-α. And after NF-κB is activated, it induces the expression of other pro-inflammatory genes including cytokines, chemokines, and adhesion molecules. This process is thought to be critical for initiating cell proliferation during tendon healing [23,51]. After a tendon injury, inflammatory cells such as neutrophils and macrophages release a variety of inflammatory cytokines including IL-1 and TNF-α, which subsequently bind to TLR4 on the cell membrane [35]. The polymerization of TLR4 enables signal transduction into cells, and there is a TIR region (Toll/IL-1 Receptor region) in the cell membrane of TLR4, which binds to the carboxy terminus of MyD88 [36]. At the same time, the amino-terminal death domain of MyD88 binds to the amino-terminal death domain of IRAK. This process promotes the phosphorylation of IRAK and the acquisition of free IRAK1, IRAK2, and IRAK4, which in turn activates TRAF-6. Next, TRAF-6 binds to NF-κB kinase and phosphorylates the beta subunit of NF-κB kinase (IKKβ), thereby activating the IκB kinase (IKK) complex [37]. IKK induces IκB phosphorylation at residues Ser32 and Ser36 of IκBα and residues Ser19 and Ser23 of IκBβ through the 26 S proteasome [38]. IκB is subsequently degraded, which results in the release of NF-κB activity and the entry of p50-p65 into the nucleus to initiate the expression of downstream genes regulated by NF-κB [39,40].

During tendon healing, NF-κB-mediated inflammatory responses often persist and overdrive the production of pro-inflammatory cytokines, including IL-1, IL-6, CCL2, and TNF-α. Fibroblasts are stimulated by these cytokines to proliferate and synthesize collagen fibers, the major component of the extracellular matrix [41]. At the same time, fibroblasts are also stimulated to transform into myofibroblasts. Myofibroblasts produce αSMA + myofibroblasts and F4/80 + macrophages that produce adhesion G protein-coupled receptors, both of which drive scar tissue formation [13]. In addition, studies have shown that the expression of p65, an important subunit of the NF-κB complex, is elevated in adhesion tissues, suggesting that the inflammatory response regulated by the NF-κB signaling pathway is closely related to the formation of fibrotic adhesions in tendon repair [51]. In the current research, some methods have been found to effectively inhibit the formation of adhesions, such as injection of IL-10 or hyaluronic acid, but the specific molecular mechanism and how to better apply them to clinical treatment still need to be explored [56].

### 3.2 Non-canonical NF-κB activation pathway

The non-canonical NF-κB pathway selectively responds to a specific set of stimuli, including ligands for subsets of TNFR superfamily members, such as LTβR, BAFFR, CD40, and RANK [57]. In the process of pathway activation, IKKα is first activated by NF-κB-inducing kinase (NIK) and then activated IKKα induces phosphorylation of P100. Processing of p100 promotes the production of p52, while the heterodimer RelB/p52 is also activated [58]. The non-canonical NF-κB complex p52/RelB translocates into the nucleus and binds to specific sites on DNA to play a regulatory role. The canonical NF-κB activation pathway is involved in almost all aspects of the immune response, while the non-canonical NF-κB pathway complements the canonical NF-κB pathway, which mainly acts on immune cells [59], such as promoting the growth and development of B lymphocytes and regulating T-cell differentiation and effector functions, thereby synergistically regulate specific functions of the adaptive immune system [55,60,61].

### 3.3 Hypoxia-induced NF-κB activation pathway

The third activation pathway is often referred to as “ non-canonical” and involves the phosphorylation of IκB by kinases such as casein kinase II or tyrosine kinase due to stress such as UV light, hypoxia, or reactive oxygen species (ROS) [47]. Hypoxia is one of the most fundamental environmental stresses experienced by cells which plays an important role in the early stages of tendinopathy. The cellular hypoxic environment that develops after a tendon injury is thought to be a potential mechanism for the incidence of degenerative tendinopathy [62]. Following tendon injury, reduced tissue perfusion and increased energy demands lead to a lack of oxygen and nutrients in the local tissue, which in turn creates a hypoxic environment [63]. In tenocytes, hypoxia can induce the expression of key cytokines and pro-inflammatory molecules, including platelet-derived growth factor, IL-6, IL-8, and platelet-activating factor, which may ultimately disrupt the balance of ECM repair [64]. Increased mechanical loading leads to apoptosis of tenocytes, whereas degradation of HIF-1α subunits is inhibited under hypoxia, thus inducing increased expression of HIF-1α during the periodic strain of tenocytes and subsequent translocation into the nucleus to regulate multiple transcription of genes [65].

HIF-1 is a heterodimer composed of two subunits, HIF-1α and HIF-1β. HIF-1α is ubiquitous in many cells and plays an important role in the intracellular hypoxia response [66]. During hypoxia, the activity of hydroxylase (PHD/FIH) is inhibited, which in turn activates the NF-κB signaling pathway [67]. At the same time, the activated NF-κB pathway also leads to the up-regulation of HIF mRNA levels, which further promotes the activation of the signal. While HIF-1 can induce the expression of NF-κB target genes, including cyclooxygenase-2 (COX-2), TNF-α, IL-6, and macrophage Phagophageal inflammatory protein-2 (MIP-2), which ultimately leads to the continued development of inflammation [67]. In another study, tyrosine phosphorylation of IKBα was shown to be an important step before its degradation from NF-κB. In contrast, HIF-1α can induce tyrosine phosphorylation of IκB by Ras/Raf kinase downstream of Src, a process similar to the typical pathway in NF-κB gene transcription [68]. In addition, mitochondria-mediated ROS generation during hypoxia may also be responsible for the activation of NF-κB [69]. In conclusion, although NF-κB is a hypoxia-responsive transcription factor, multiple specific activation mechanisms may exist.

## 4 Role of NLRP3 signaling pathway

**Figure 4:**
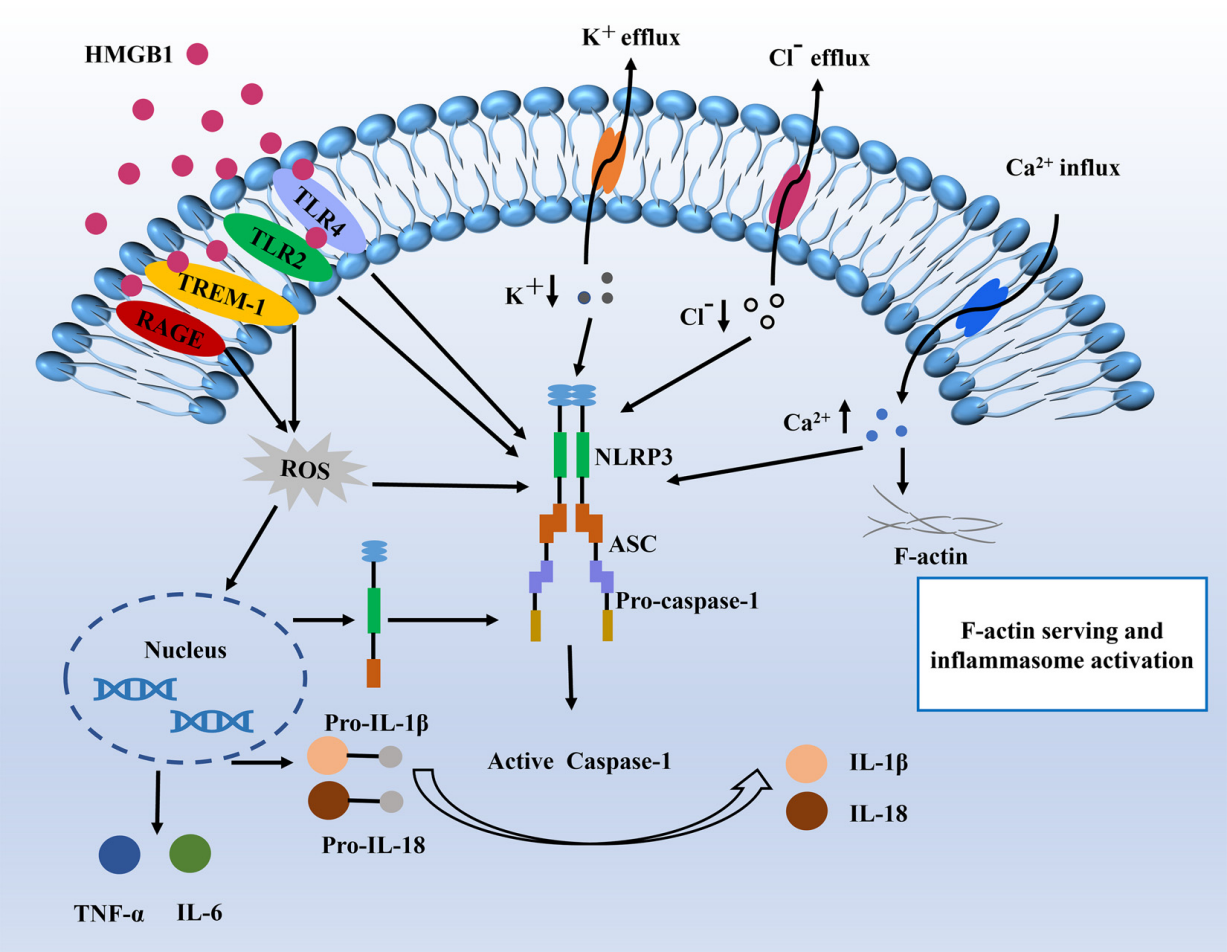
Process of NLRP3-induced inflammatory response.

The inflammasome is a multi-protein signaling complex that recognizes pathogenic-associated molecular patterns (PAMPs) and damage-associated molecular patterns (DAMPs), and thus is widely involved in the pathogenesis of various diseases [70]. Among the various inflammatory vesicle complexes, the NLRP3 inflammatory vesicle complex is the most common one in the inflammatory phase of tendinopathies. It consists of the sensor NLRP3, the adapter ASC, and pro-caspase-1 [71]. NLRP3 can respond to various exogenous or endogenous danger signals, including various microbial products, endogenous molecules, and changes in intracellular ions, which can activate NLRP3. This promotes the release of a range of cytokines that contribute to the development of an inflammatory response [72]. Therefore, NLRP3 plays an important regulatory role in the inflammatory mechanism of tendinopathy. After a tendon injury, HMGB1 released by necrotic cells becomes the main pathway of NLRP3 activation. HMGB1 can ultimately trigger the assembly and activation of the NLRP3 inflammasome by activating multiple receptors, including TREM-1, TLR4, Toll-like receptor 2 (TLR2), and RAGE [24]. Following activation of NLRP3 promotes the release of cytokines and induces ECM disturbances and increased inflammatory responses, ultimately hindering the process of tendon healing. In addition, changes in various cellular signals can also lead to NLRP3 inflammasome activation, such as K^+^ efflux, Ca^2+^ signaling, and mitochondrial dysfunction. However, the specific mechanisms of action between them remain to be studied [73]. The process by which NLRP3 induces an inflammatory response is depicted in Figure 4.

HMGB1 binds to a series of receptors on the cell membrane surface, including RAGE, TLR2, TLR4, and TREM-1. Upon binding, NLRP3 is activated. NLRP3 promotes the aggregation of apoptosis-associated speckle-like protein (ASC), subsequently recruits caspase-1, and facilitates its processing. After conversion, active caspase-1 is formed, which finally converts cytokines pro-IL-1β and pro-IL-18 into mature and biologically active IL-1β and IL-18. The increase in Ca2 + enabled FliI to inhibit the F-actin function. F-actin was unable to interact with FliI and leucine-rich repeat FliI-interacting protein 2 (LRRFIP2), so the inhibition of NLRP3 was relieved. The subsequent enhanced activity of NLRP3 is involved in promoting the maturation and release of IL-1β.

### 4.1 Molecular mechanism of HMGB1-stimulated NLRP3 inflammasome activation

In the early stages of tendinopathy, cell necrosis or stimulation by stressful conditions (hypoxia, mechanical) leads to the release of endogenous danger signals or alarmins, resulting in the activation of an immune response. This process is called damage-associated molecular patterning (DAMP) [74]. Alarmin, a member of DAMPs, is also a key point in triggering the onset and ongoing development of the inflammatory response in tendinopathy. Various alarmins have been reported in tendinopathy, including HMGB1, IL-1α, and IL-33. Studies have shown that HMGB1 is closely related to the activation of NLRP3 [75].

HMGB1 is widely expressed in organisms and is a highly conserved nuclear protein that can be passively released from necrotic cells or actively secreted from immune cells activated by inflammatory factors [76]. After HMGB1 in the nucleus is secreted to the outside of the cell, it binds to a series of receptors on the cell membrane surface, including the receptor for advanced glycation end products (RAGE), TLR2, TLR4, and TREM-1 [77]. After binding, it is involved in downstream signaling molecules in transcriptional regulation and triggers the activity of NLRP3. NLRP3 promotes the aggregation of ASC, subsequently recruits caspase-1 and facilitates its processing. After conversion, active caspase-1 is formed, which finally converts cytokines pro-IL-1β and pro-IL-18 into mature and biologically active IL-1β and IL-18 [78].

The RAGE is one of the ligands of HMGB1. The binding of HMGB1 to RAGE in inflammatory cells promotes cell migration and angiogenesis, ultimately leading to the recruitment of more inflammatory cells and the release of mediators [79]. At the same time, as a ligand of TLR2 and TLR4, HMGB1 can also have a synergistic effect on NLRP3-mediated inflammation [70]. In tendinopathy tissue, elevated levels of HIF-1α indicate a hypoxic and ischemic environment at the injury site [66]. In this environment, HMGB1 is released into the extracellular space in a full thiol state and acts on the Ig superfamily receptor RAGE, which binds to advanced glycation end products to activate downstream signaling and generates ROS to activate NLRP3, eventually triggering a series of inflammatory responses [80,81]. However, Cys-106 of HMGB1 remains in the –SH (thiol) state to activate TLR4, while Cys-23 and Cys-45 of HMGB1 form a disulfide bond, which interacts with TLR4 [82]. In addition, HMGB1 can also activate TLR2 signaling. Subsequent ligand binding of TLR4 and TLR2 induces the secretion of IL-1β and finally promotes the assembly of NLRP3 [83]. Tenocytes are able to secrete TREM-1 together with neutrophils and macrophages [24]. Like TLRs and RAGEs, TREM-1 is a receptor for HMGB1 .In hypoxia, activation of immunoglobulin receptor superfamily member myeloid cell 1 (TREM-1) through upregulation of DAP12 and NF-κB activity can also activate NLRP3 activity [84]. Activation of TREM-1 stimulates intracellular Ca^2+^ efflux, which subsequently leads to mitochondrial Ca^2+^ overload and ROS generation [85]. Studies have shown that ROS is another effective activator of the NLRP3 inflammasome, so TREM-1 is closely related to the activation of the NLRP3 inflammasome. However, since the role of TREM-1 in inflammation is an emerging field, the underlying mechanism of action remains to be further investigated.

### 4.2 Other non-canonical pathways activate NLRP3 activity

In addition to the above-mentioned common NLRP3 activation pathways, there are some non-canonical activation pathways. One study found a decrease in tendon COLⅠ:COLⅢ ratio consistent with an increase in NLRP3 expression during the ECM remodeling phase after rotator cuff injury [15]. And it has been reported that in bone tissue, the degradation of the bone matrix can activate NLRP3 [86]. Therefore, we speculate that the disorder of ECM is closely related to the expression of NLRP3 in tendon tissue. However, the specific mechanism of action is still unclear.

In addition, studies have shown that persistent adipose tissue infiltration in tenocytes hinders tendon healing by prolonging the inflammatory response [87]. However, the synergy between TLR2, TLR4, TREM-1, and RAGE was found in hepatocytes to induce upregulation of HMGB1 and promote adipose tissue infiltration [88]. Inhibition of NLRP3 activity prevented adipose tissue accumulation in hepatocytes, suggesting a potential role of NLRP3 in promoting adipose tissue infiltration in tissues [89]. Although no relevant reports have been found in tenocytes, we can still speculate that the infiltration of adipose tissue in tenocytes is related to the activation of NLRP3.

Intracellular flux of multiple ions, including K^+^, Ca^2+^, and Cl^−^, has been reported to promote NLRP3 inflammasome activation [90]. F-Actin can interact with a calcium-dependent actin remodeling protein, Flightless-I (FliI), and an NLRP3-related protein, LRRFIP2. And this effect can inhibit NLRP3 inflammasome activation [91]. However, cyclic stretching induces an increase of Ca^2+^ in tenocytes, which in turn leads to F-actin depolymerization, while the increase in Ca^2+^ allows FliI to inhibit F-actin function. F-actin was unable to interact with FliI and LRRFIP2, so the inhibition of NLRP3 was relieved [92]. The subsequent enhanced activity of NLRP3 is involved in promoting the maturation and release of IL-1β, which aggravates inflammation outside the tenocytes.

Changes in K^+^ concentration are also considered to be an important factor in the activation of NLRP3. One study showed that the use of high concentrations of K^+^ extracellularly can directly block the activity of NLRP3 [93]. At the same time, high concentrations of extracellular K^+^ can also play a role in the upstream signaling pathway activated by NLRP3, inhibiting the activation of NLRP3 inflammasome by various stimuli [73]. However, the specific link between K^+^ concentration and NLRP3 activity still needs to be further explored.

## 5 Role of p38/MAPK signaling pathway

MAPKs are a class of protein kinases specific for serine, threonine, and tyrosine, and they are important signaling pathways in living organisms [94]. They are first activated by a series of extracellular stimulatory signals and change the phosphorylation state, then interact with upstream kinases, downstream substrates ,and phosphatases, and finally transmit signals from the cell membrane to the nucleus [95]. In tendinopathy tissues, they regulate many physiological and biochemical activities, including secretion of inflammatory factors, synthesis and degradation of ECM, and apoptosis [96]. The MAPK family has four members, including extracellular signal-regulated protein kinases (ERKs), p38/MAPK, c-Jun amino-terminal kinase/emergency-activated protein kinase (JNK/SAPKs), and ERK5/megafilament lysin-activated protein kinase (BMK1) [97].

**Figure 5:**
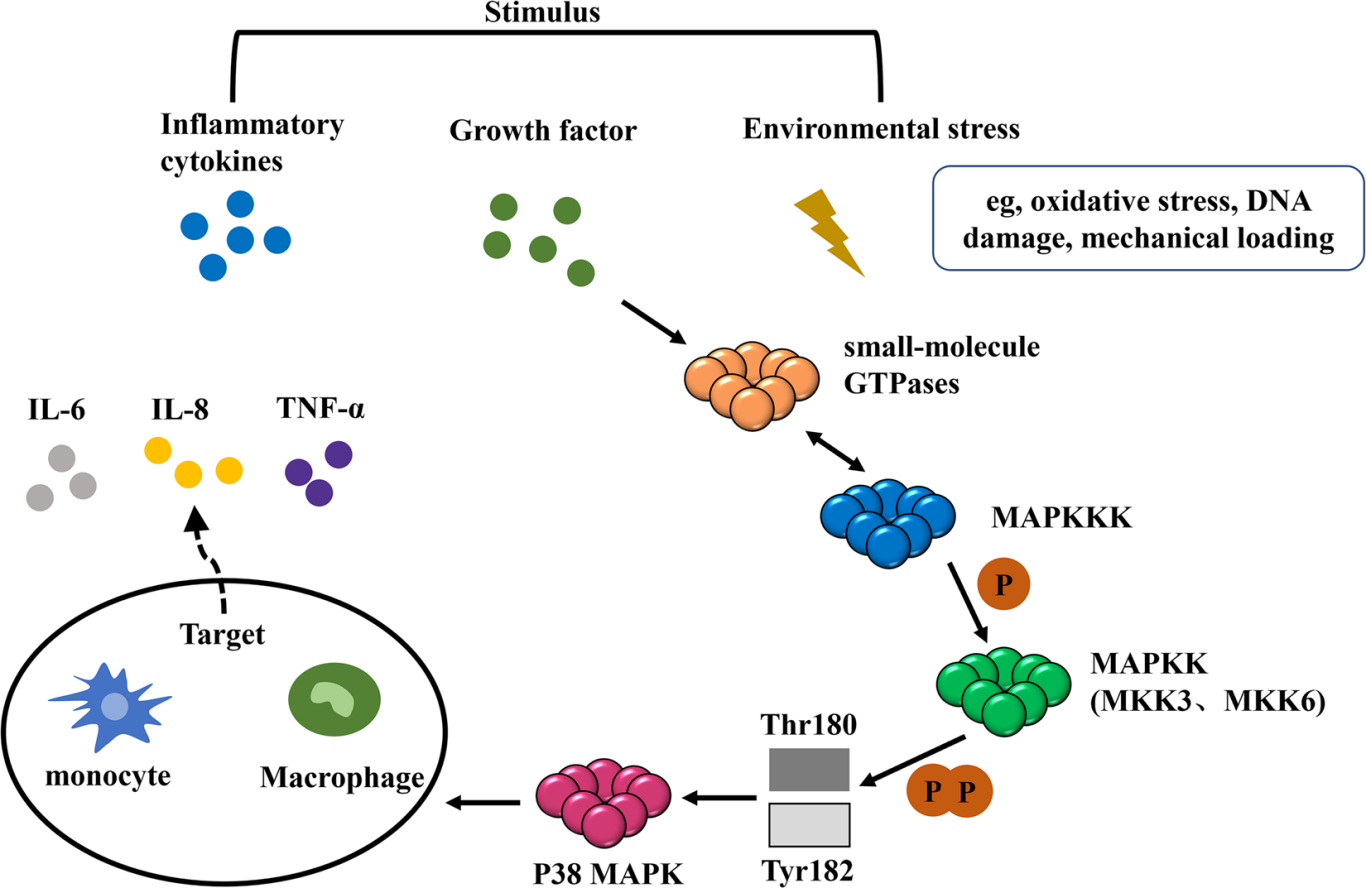
Inflammatory response process involved in p38MAP.

p38/MAPK is one of the important members of MAPKs; it has four subtypes, including p38α, p38β, p38γ, and p38δ. p38/MAPK is activated when the body is under stress or mechanical stimulation, and it plays an important role in physiological activities such as inflammation, cell growth and development, and apoptosis [98]. Especially, the role in inflammation is the most prominent aspect [99]. Signaling of the p38/MAPK pathway is typically activated through interactions between upstream components of the activation cascade and adaptor proteins. This process usually takes three to five kinase phosphorylations to transmit the signal smoothly. Three core kinases are required in this process, including MAPK kinase kinase (MAPKKK), MAPK kinase (MAPKK), and MAPK [100]. Small-molecule GTPases are often activated after stimuli by inflammatory factors, growth factors, or environmental stress (e.g., oxidative stress, DNA damage, and mechanical loading). MAPKKK usually completes its activation by interacting with small-molecule GTPases. Subsequently, activated MAPKKK directly phosphorylates and activates MAPKK [101]. There are mainly two types of MAPKK in the p38 signaling pathway, MKK3 and MKK6. Next, MKK3 and MKK6 doubly phosphorylate Thr180 and Tyr182 and activate MAPK [102]. Once activated, MAPK acts on downstream targets, thereby promoting the production of inflammatory factors such as TNF-α, IL-6, and IL-8 by monocytes and macrophages and finally regulating the inflammatory response [94]. The inflammatory response process involved in p38/MAPK is shown in Figure 5.

It has also been found that the pro-inflammatory state generated by the enhanced activity of NF-κB and p38/MAPK is one of the bases for the development of heterotopic ossification (HO)[103]. And it was observed in *in vitro* experiments that the addition of (p38) MAPK inhibitor SB203580, (ERK) MAPK inhibitor PD98059, and (JNK) MAPK inhibitor SP600125 to the cells resulted in a decrease in the degree of phosphorylation of ERK, JNK, p38, NF-κB, and IKKβ, as well as a significant reduction in the degree of HO[104]. These results suggest that the inflammatory response can be significantly attenuated by inhibiting the activity of MAPK and have a therapeutic effect on HO. However, there is no sufficient data about *in vivo* experiments in humans, so in the future, we need to conduct more relevant experiments in depth to explore how MAPK inhibitors can be more safely and effectively applied to attenuate the inflammatory response in human tendinopathy.

The cytokine first activates the small molecule GTPase, and subsequently, MAPKKK is activated by interacting with the small molecule GTPase. Subsequently, the activated MAPKKK directly phosphorylates and activates MAPKK. Next, MKK3 and MKK6 doubly phosphorylate Thr180 and Tyr182 and activate MAPK. When MAPK is activated, it acts on downstream targets and promotes the production of inflammatory factors by monocytes and macrophages.

## 6 Role of STAT3 signaling pathway

STAT is the main signaling pathway in the body, which consists of STAT and its receptors. The STAT protein family is a group of related proteins that can be activated by different cytokine receptors [105]. Among them, STAT3 is an important member of the STAT family, which plays an important role in the body’s immune response, cell proliferation and differentiation, angiogenesis, apoptosis, as well as tumorigenesis [106]. Interferons, interleukins, growth factors, and other chemical signals bind to cell surface receptors and activate associated kinases. The activated kinase subsequently phosphorylates itself and the receptor, while also phosphorylating tyrosine residues of the downstream target protein. After the earlier process, the transcription factor STAT3 is recruited and phosphorylated, and then, it enters the nucleus in the form of a dimer to bind to target genes and regulate the transcription of downstream genes [107].

**Figure 6:**
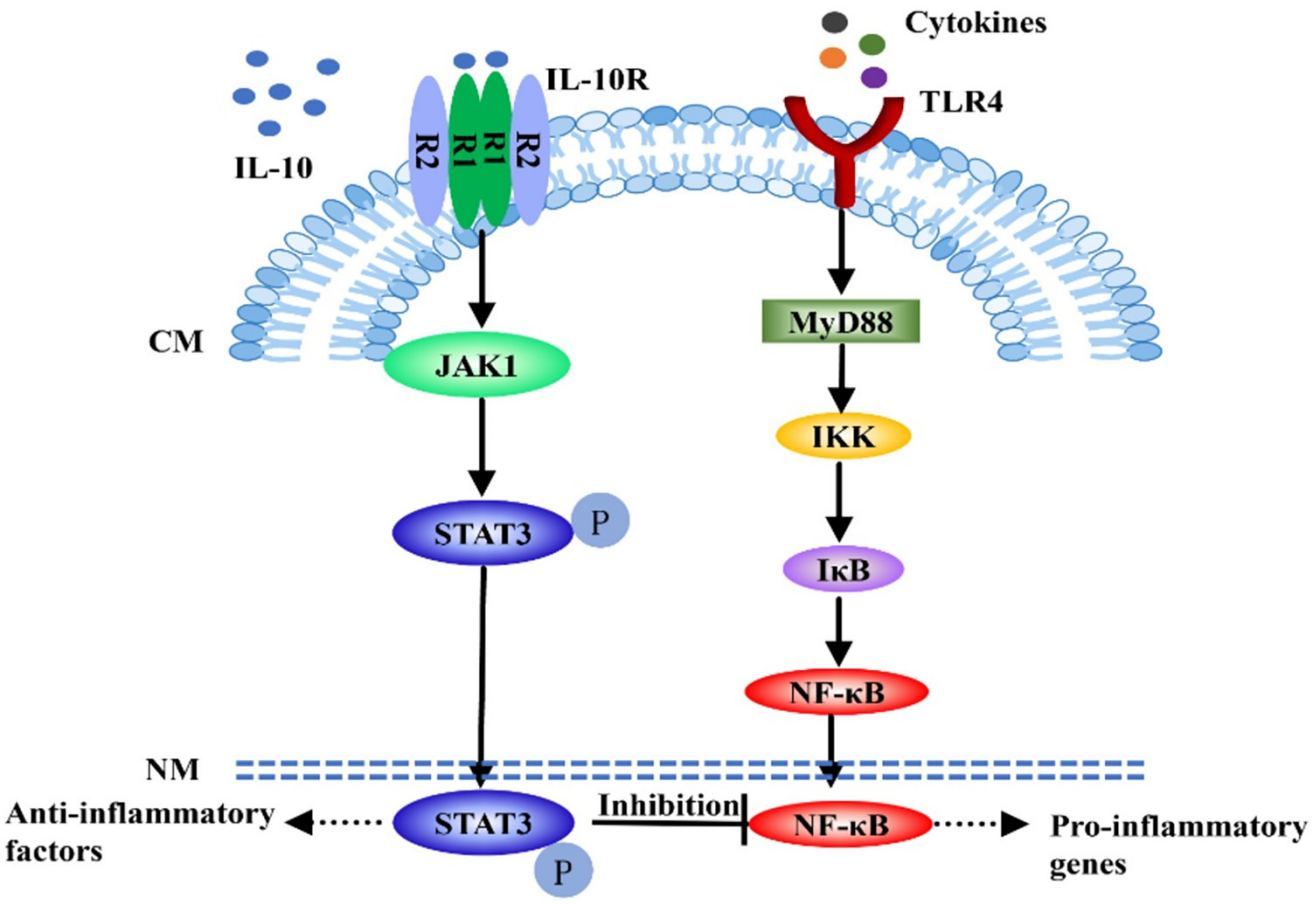
IL-10 Exerts anti-inflammatory effects through STAT3 signaling pathway.

In the inflammatory response of tendinopathy, STAT3 has a dual regulatory role, but the specific mechanism of action is still unclear. On the one hand, studies have shown that after reducing the use of JNK/STAT3 signaling inhibitors, the expression of the proinflammatory factor IL-6 is significantly increased, and the IL-6-induced inflammatory response can promote wound healing through fibrosis and scarring [108]. However, persistent inflammation often leads to excessive proliferation of myofibroblasts and promotes excessive deposition of ECM components at the site of injury, leading to scar tissue formation [109]. In addition, studies in recent years have shown that the JAK/STAT signaling pathway is a novel mechanism leading to tendon stem/progenitor cells (TSPC) senescence. Chronic sterile inflammation is a hallmark of aging, and the JAK/STAT signaling pathway mediates the expression of many pro-inflammatory cytokines, matrix metalloproteinases, and chemokines, and leads to persistent chronic inflammation. Therefore, inhibition of the JAK/STAT signaling pathway may be an ideal target to alleviate the inflammatory response after tendon injury as well as tendon senescence [110]. On the other hand, the STAT signaling pathway is necessary for IL-10 to exert anti-inflammatory effects as well as to promote IL-10 production [108]. For example, IL-10 regulates the TLR4/NF-κB signaling axis in dermal fibroblasts via the IL-10R/STAT3 signaling pathway, thereby reducing the inflammatory response and ultimately preventing scar formation [56]. Specifically, IL-10 forms homo- or heterodimers by binding to the homologous receptor IL-10R (IL-10R is a tetramer containing two IL-10R1 polypeptide chains and two IL-10R2 chains), which then activates the receptor-linked tyrosine protein kinase 1 (janus kinas 1, JAK1) [111]. JAK1 subsequently phosphorylates tyrosine residues (Y446 and Y496) specific to the intracellular structural domain located in IL-10R1. Once phosphorylated, these tyrosine residues provide a temporary anchor site for the potential transcription factor STAT3. STAT3 binds to these sites via its SH2 structural domain, and activated STAT3 then leaves the receptor and forms homo- or heterodimers in the cytoplasm. The dimer then rapidly moves into the nucleus, binds to its specific DNA sequence, and initiates intranuclear DNA transcription. In the following process, an anti-inflammatory cascade begins, including inhibition of NF-κB activity and inhibition of its ability to bind DNA [112]. The specific mechanism by which IL-10 exerts its anti-inflammatory effects through the STAT3 signaling pathway is shown in Figure 6. It has also been shown that after stimulation of tendon stem cells (TSCs) with connective tissue growth factor, the production of the anti-inflammatory cytokine IL-10 was induced through the JNK/STAT3 signaling pathway, which effectively reduced the inflammatory response, ECM secretion, and scar tissue [17]. Similarly, it was shown that JNK/STAT3 signaling pathway plays a key role in aspirin-induced IL-10 and TIMP-3 expression in TSCs. Aspirin upregulates the expression of the anti-inflammatory factor IL-10 through the JNK/STAT3 signaling axis, thereby impeding IL-1β-induced migration and proliferation of TSCs. In turn, the decrease in TSCs number may reduce the deposition of ECM in the injured tendon, thereby reducing scar formation. Thus, aspirin may indirectly improve tendon healing by regulating inflammation through the JNK/STAT3 signaling pathway [108]. In addition to pharmacological interventions, some valuable research results have been obtained in recent years in the direction of using mechanical stimulation to modulate the inflammatory response. For example, mechanical stimulation of tendons by running on a treadmill has been shown to increase the expression of IL-4 at the tendon-bone junction, and the binding of IL-4 to its receptor activates JAK1 and JAK3, which in turn leads to the activation of STAT and its translocation to the nucleus. Ultimately, activation of the JAK/STAT signaling pathway shifts macrophages from a pro-inflammatory to an anti-inflammatory phenotype, resulting in the production of a series of anti-inflammatory cytokines that regulate the local inflammatory microenvironment to promote tissue healing [113]. However, more in-depth studies, such as sequencing analysis, are needed in the future to explain the specific mechanisms and targets of the overall inflammatory regulatory process. Therefore, STAT3 plays a crucial role in regulating the balance of inflammation and ECM during the healing of injured tendon.

Although there is a growing interest in using the STAT3 signaling pathway to reduce inflammatory responses in tendinopathies, there are still many issues that need to be further investigated, such as the lack of an accepted standard for the dose or duration of drug intervention in many trials, which is therefore still a long way from being applied in clinical trials. In addition, more in-depth studies are needed to clarify the mechanism of action and specific targets for some of the newly explored pathways.

IL-10 participates in anti-inflammatory responses through the IL-10R/STAT3 signaling pathway, including inhibition of NF-κB activity and inhibition of its ability to bind DNA.

## 7 Conclusion and perspectives

This review summarizes several major pathways by which the inflammatory response occurs by discussing the mechanisms of action of several major signaling pathways associated with inflammation in tendinopathies and provides new ideas for inhibiting the ongoing inflammatory response and treating tendinopathy.

Although there has been a lot of important research on tendon repair, the research in the inflammatory stage still needs to be further explored. In particular, the signaling pathways closely related to the inflammatory response maintain the homeostasis of tissues during the inflammatory response by regulating the transcription of inflammatory factors. The inflammatory response is critical for effective tendon repair. Only by initiating an inflammatory response to promote the release of cytokines and growth factors can necrotic material and ECM debris be removed and the proliferative and remodeling phases of the healing process proceed smoothly [12]. However, a persistent inflammatory response can also have negative effects. If the inflammatory response in the early stage of tendon repair continues unabated, it will lead to excessive recruitment of pro-inflammatory mediators; especially, the transcription of pro-inflammatory mediators induced by the NF-κB signaling pathway is considered to be a key regulator of the inflammatory response [44]. A persistent inflammatory environment induces increased myofibroblast migration and production, promotes excessive collagen synthesis, and leads to excessive deposition of ECM, resulting in an excessive “fibrotic repair response” [114]. A dense layer of connective tissue called penitential fibrotic adhesion eventually forms between the tendon and surrounding tissue, and this fibrotic change is also thought to be an important factor in the subsequent development of chronic degenerative tendinopathy [115,116]. Fortunately, many studies have now shown that by applying specific inhibitors of the inflammatory pathway, the activity of the inflammatory pathway can be significantly inhibited, thus reducing the inflammatory response. However, there are still many limitations in the current application. For example, the number of human case samples currently used in clinical trials is small, and there are no clear standards for the dosage of pathway-specific inhibitors to be administered and the duration of treatment, which need to be determined by further experiments[51]. In addition, many small molecule inhibitors are delivered systemically, which may not be an ideal approach for the treatment of localized inflammation. Therefore, we need further exploration of the mode of drug delivery[50]. For some natural drugs that have the same effects as inhibitors, although phase I clinical trials have shown no adverse effects in human subjects at high doses, the effects of these natural drugs on humans need to be studied at the cellular and molecular levels to ensure the safety and efficacy of the application of these drugs [117]. Several recent studies have delved into some new inflammatory pathways in tendinopathies, which have great potential for targeted treatment of inflammation. In the studies of mast cells, it was found that mast cells can express glutamate receptors after tendon injury, and glutamate receptors are the main substances that mediate neuronal inflammation in tendinopathies [118]. Therefore, in future studies, targeted inhibition of mast cells and interference with the activation of glutamate receptors are new strategies to attenuate the inflammatory response. In addition, it was found that the receptor agonist of adenosine A2A, polydeoxyribonucleotide (PDRN), could activate the cAMP–PKA–CREB pathway and reduce the percentage of caspase-3-positive cells and caspase-9-positive cells in rat Achilles tendon injury, thereby inhibiting the secretion of pro-inflammatory cytokines. Therefore, PDRN injection has a satisfactory effect in anti-inflammation and inhibition of apoptosis [119]. Therefore, a comprehensive and in-depth understanding of the signaling pathways of the inflammatory response is crucial to remove the harmful part of the inflammatory cascade at the right time. At the same time, it is of great significance for the development of new anti-inflammatory drugs. Although there are many anti-inflammatory drugs previously used in clinical treatment, such as non-steroidal anti-inflammatory drugs and corticosteroids, the effect is not satisfactory. Although these anti-inflammatory drugs show beneficial effects in the short term, long-term use may impair the structural integrity of the tendon and increase the risk of tendon rupture in many studies [120,121].

In recent years, a number of new biomaterial-based therapeutic approaches have attracted attention and shown satisfactory results in the anti-inflammatory treatment of tendinopathies. For example, synthetic polymer scaffolds have better mechanical stability and can mimic the arrangement structure of collagen fibers in tendons, which can help cells at the site of injury transition from the inflammatory microenvironment to the regenerative microenvironment [122]. However, since some materials of stents have a high degradation rate, such as polylactic acid (PLA) and polyglycolic acid (PGA), which can lead to an enhanced inflammatory response, they are more suitable for the repair of microscopic injuries [123]. However, scaffolds prepared from natural biomaterials are usually well suited to promote cell adhesion and thus play an important role in facilitating tendon repair. For example, scaffolds made of biomaterials such as hyaluronic acid or chitosan have the effect of preventing inflammatory cells from adhering to fibronectin, thereby reducing the inflammatory response [124]. However, the immunomodulatory effects they mediate usually show dependence on molecular weight and applied dose. Therefore, their effect on immune cell activity and the variation of these factors in tendon repair needs to be studied in more depth in the future [125]. There is also an emerging treatment method that is also attracting attention, and that is hydrogel. It has been shown to provide mechanical support to injured tendon tissue and to treat tendon injuries by means of sustained drug release. This approach is useful for regulating the secretion of inflammatory factors and promoting the polarization of macrophages toward the anti-inflammatory phenotype. This method also has some disadvantages, such as lack of toughness and burst release of the drug [126]. Therefore, more in-depth research is needed in the future to correct these shortcomings.

In recent years, an increasing number of findings have shown the critical role of the inflammatory response in tendinopathies. New ideas are also being introduced in the study of tendinopathy inflammation, such as the use of single-cell sequencing as well as spatial transcriptomics to identify key cellular phenotypes driving pathogenesis and thus target these pathogenic cytokines and signaling pathways [127]. Therefore, an in-depth understanding of the mechanism of action of inflammatory signaling pathways is crucial for the development of safe and easily controlled treatments to improve tendon healing.
